# Kinetic and Thermodynamic Analyses of Co-Pyrolysis of Nylon-Polyethylene Pouch Wastes

**DOI:** 10.3390/ma16175738

**Published:** 2023-08-22

**Authors:** Hai-Bo Wan, Zhen Huang

**Affiliations:** Tianjin University of Commerce, Tianjin 300134, China; wanhaibo@tjcu.edu.cn

**Keywords:** plastic wastes, co-pyrolysis, thermogravimetric analysis, isoconversional kinetics

## Abstract

In this study, thermogravimetric measurements of nylon-6/polyethylene double-layer pouch wastes were conducted in N_2_ under a constant heating-rate mode, and the multiple heating-rate results were analyzed in terms of degradation features and specific temperatures. Experimental results show that the waste pyrolysis involves one reaction stage, and all specific parameters appear to increase with the heating rate. Kinetic analysis of non-isothermal data was thoroughly performed using various isoconversional model-free methods for the calculations of the activation energy, resulting in 143~215 kJ/mol over the whole pyrolysis process. By means of the model-fitting method, the reaction mechanism model *g*(*α*) and pre-exponential factor ln*k*_0_ are concurrently determined with the aid of the linear compensation effect. With such methodology proposed, the Avrami–Erofeev kinetic model A_3/2_ of *g*(*α*) = [−ln(1 − *α*)]^2/3^ is found to be the most appropriate mechanism function for describing the pyrolysis of the nylon-6/polyethylene waste along with ln*k*_0_ of 23.14 to 34.26 min^−1^. With the Arrhenius parameters thus obtained, the predictions were made and performed very satisfactorily to correlate experimental results. Additionally, the service life and thermodynamic parameters over the entire pyrolysis process were also estimated.

## 1. Introduction

Today, plastic films are widely applied in the food packaging industry such as various flexible packaging articles, liners inside cartons and lidding on cups. In these usages, plastic films can be employed alone or in combinations to serve as diverse packages for various fresh produce. Thus, these films should satisfy the industry requirements and consumer desires so as to maintain food safety and minimize environmental damage. Therefore, polymers used to make packaging plastic films are required to balance the packaging permeability requirements, the respiratory activities of the fresh foods and the surrounding temperature and humidity. Among these plastic films used, nylon-6/polyethylene (N-PE) double-layer films appear very attractive for preserving fresh foods [1,2] since their mechanical, thermal and gas-permeable properties complement each other [3]. With the rapid development of the food packaging industry and the demand for plastics, the accumulation of plastic waste has increased, and the environmental hazards thus caused have gained widespread concern around the world. Among various plastic waste disposal technologies, pyrolysis appears to be notable because it can recover value-added chemical species from plastic wastes and efficiently minimize environmental risks [4].

For the purpose of studying pyrolysis processes, thermogravimetric analysis (TGA) is known to be the most widely used method [5,6,7]. Along with experimental attempts, theoretical kinetic analysis of the plastic pyrolysis process has also played a vital role in guiding the pyrolysis reactor design and providing theoretical support for industrial application. Based on TGA data over different heating rates, model-free and model-fitting methods are two parallel ways for performing kinetic pyrolysis analysis in terms of kinetic triplet parameters of apparent activation energy *E_k_*, pre-exponential factor *k*_0_ and reaction mechanism model function *f*(α) [8,9,10]. By means of the model-free method, the activation energy can be more reliably resulted because there is no involvement of any pyrolysis mechanism assumption. In contrast, model-fitting methods can be attempted for obtaining both the pre-exponential factor and the pyrolysis reaction mechanism function. Usually, the study of pyrolysis behavior and kinetic analysis has been performed on individual plastics since it aids to comprehend the pyrolysis of plastic blends [5,6].

In the current study, nylon-6/polyethylene (N-PE) double-layer film pouch wastes were collected locally in Tianjin, China, for inert pyrolysis decomposition considerations. Up to now, many efforts have been made to individually investigate the pyrolysis of nylon–6 [11,12,13,14] and polyethylene [15,16,17,18] so as to provide necessary knowledge for thermochemically conversing their wastes into valuable chemical compounds for sustainable energy recovery. These kinetic works have shown that for the pyrolysis of nylon–6 [11,12,13,14], it has *E*_k_ = 113~266 kJ/mol and ln*k*_0_ = 8.7~15.0 min^−1^ while its one-step decomposition may be controlled by the order-based chemical reaction Fn model of n = 0.33~1.9. As for the pyrolysis of PE [15,16,17,18], the *E*_k_ and ln*k*_0_ values are found to vary between 135 and 250 kJ/mol, or 3.32 × 10^17^~2.20 × 10^21^ min^−1^, respectively, while the one-step pyrolysis appears to be also controlled by the order-based chemical reaction models of the F_1/2_ model with *g*(*α*) = 1 − (1 − *α*)^1/2^ or the F1 model with *g*(*α*) = −ln(1 − *α*). However, Budrugeac et al. [7] proposed a very complex LDPE pyrolysis composed of four successive stages: Avrami–Erofeev kinetic models (An), reaction order (Fn), reaction order (Fn), and three-dimensional diffusion (D3). Obviously, these kinetic parameters are different from diverse works and somewhat disagreeable with each other. The main reason for it may be related to the different sources of raw samples, different preparation techniques and the introduction of fillers, experimental programs and instrument limitation conditions [7].

The main contributions of the present work are as follows: (1) Pyrolysis features and kinetics of N-PE waste in inert nitrogen were fully obtained by using a thermogravimetric analyzer. To our best knowledge, there is no report on the pyrolysis investigation of the nylon-6 and PE mixture, and such work is required not only to get information about pyrolysis features and kinetic parameters over the whole temperature range but also to provide pyrolysis process predictions necessary for thermochemically processing massive N-PE waste. (2) Non-isothermal mass loss data were kinetically analyzed to determine the activation energy with various model-free methods, and the calculation results with these methods were compared. (3) A new compensation-effect-aided model-fitting method was attempted to determine the most appropriate mechanism function for describing the pyrolysis process of N-PE waste and the pre-exponential factor as well. (4) With the kinetic quantities, the predications against experimental mass loss results along with the lifetime estimation were thus made. In addition, thermodynamic parameters were also computed over the entire pyrolysis process according to the transition state theory. The present work provides information about pyrolysis behaviors and kinetic parameters necessary for designing any chemical reactor to thermally dispose of N-PE waste.

## 2. Materials and Analysis Methods

### 2.1. Materials

Nylon-6/PE (N-PE) double-layer pouch wastes, after we completed the preservation packaging test for mango fruits in our laboratory, were collected and washed using distilled water. The double-layer film to make the pouches was determined to have a thickness of about 80 μm, and it was purchased from Cangzhou Senyu Plastics Co., Ltd., Cangzhou, China, and co-extruded with nylon-6 and low-density polyethylene at a mass ratio of 1 to 3. The washed pouch waste samples, after drying at 350 K in a controlled oven for 2 h, were ground mechanically and meshed into powders of less than 100 μm in size for subsequent experiments.

### 2.2. TGA Analysis

A Shimadzu DTG-60 thermogravimetric analyzer was used to carry out thermal degradation measurements and simultaneous differential thermal analysis (DTA) under 30 cm^3^ N_2_ flow per minute. For all measurements, the N-PE waste samples were weighed to have approximately similar mass of 3~4 mg and then were subjected to heating with a temperature rise of 5, 10, 15 and 20 K per minute from ambient to 850 K. The non-isothermal mass loss data and the first derivative data of thermogravimetric curves (DTG) along with DTA data were all automatically generated and recorded via the accompanied software.

### 2.3. Theoretical Basis for Kinetic Analysis

To deeply understand pyrolysis behaviors of solid materials, kinetic analysis of thermal degradation processes is usually performed, resulting in kinetic triplets in terms of the activation energy (*E*_k_), the pre-exponential factor (*k*_0_) and the reaction mechanism function (*f*(*α*)) [8,9,10]. Theoretically, kinetic triplet parameters can be obtained by using model-free and model-fitting methods to evaluate isothermal pyrolysis data.

In general, pyrolysis process of plastic wastes may be mathematically expressed by the rate of degradation (*dα*/*dt*) depending on degradation time and temperature:

(1) dα/dt=k(T)⋅f(α)

where *α* is the degree of pyrolysis degradation defined as *α* = (*w*_0_ − *w*_t_)*/*(*w*_0_ − *w_f_*), and *w*_0_, *w_t_* and *w_f_* are the initial mass, the mass at pyrolysis time *t* and final mass of the sample, respectively. *f*(*α*) stands for any specific reaction mechanism model in its differential expression, which is commonly taken as the function of *α* only, and the *α* data could be readily abstracted from experimental TG results. *k*(*T*) is the rate constant of pyrolysis process and is usually thought to depend on absolute temperature *T*(K) following Arrhenius law:

(2) $$k(T)=k_{0}\cdot \exp(-E_{k}/RT)$$

where *k*_0_ is the pre-exponential factor (min^−1^), and *E_k_* and *R* are the activation energy (kJ/mol) and gas constant (8.314 J/mol·K), respectively.

Under non-isothermal conditions with a heating rate (K/min) defined as *β* = *dT*/*dt*, one may easily obtain

(3) $$\frac{da}{dT}=\frac{k_{0}}{\beta }\cdot \exp(-E_{k}/RT)\cdot f(a)$$

After transformation, the following may be deduced:

(4) $$g(\alpha )\equiv \int_{0}^{\alpha }\frac{d\alpha }{f(\alpha )}=\frac{k_{0}}{\beta }\int_{0}^{T}\exp\left(-\frac{E_{k}}{RT}\right)dT$$

where *g*(*α*) is the reaction model in the integral expression and stands for a particular pyrolysis mechanism function *f*(*a*). One may note that the right-hand side of Equation (4) involves a well-known temperature integral, which cannot be analytically solved but can be resolved to be either numerical solutions or diverse approximate solutions. On the other hand, a number of reaction mechanism models in *g*(*a*) or *f*(*a*) have been proposed and can be referred to elsewhere [19,20].

Therefore, kinetic triplets of *E*_k_, *k*_0_ and *f*(*α*) must be achieved for completely depicting any pyrolysis process and providing necessary knowledge for any related practical applications. Usually, the *E_k_* values can be readily calculated by using model-free methods while the *f*(*α*) may be properly judged by means of the model-fitting methodology. As for a third kinetic parameter *k*_0_, it may be determined thereafter by considering the compensation effect or fitting against experimental results. In present study, model-free and model-fitting methods were applied for kinetically recounting pyrolysis of N-PE wastes.

#### 2.3.1. Determination of E_a_ Based on Isoconversional Methods

For the determination of the activation energy *E_k_*, a number of model-free methods have been extensively attempted since they do not consider any specific chemical reaction model but are mostly developed by approximating temperature-integral functions [8,9,10,21,22]. Up to now, the most commonly used integral methods include Coats–Redfern (CR) [23], Flynn–Wall–Ozawa (FWO) [24,25], Madhusudanan–Krishnan–Nina (MKN) [26] and Starink (SK) [27] methods, and they are mathematically expressed as below:

(5) $$\mathrm{CR}\;\mathrm{method}:\;\ln\left(\frac{\beta }{T^{2}}\right)=\ln\left[\frac{k_{0}R}{E_{k}g(\alpha )}\right]-\frac{E_{k}}{RT}.$$

(6) $$\mathrm{FWO}\;\mathrm{method}:\;\ln\beta =\ln\left[\frac{0.0048k_{0}E_{k}}{Rg(\alpha )}\right]-1.0516\frac{E_{k}}{RT}.$$

(7) $$\mathrm{MKN}\;\mathrm{method}:\;\;\ln\left(\frac{\beta }{T^{1.884318}}\right)=\ln\left[\frac{k_{0}}{g(\alpha )}{\left(\frac{E_{k}}{R}\right)}^{-0.884318}\right]-1.001928\frac{E_{k}}{RT}-0.389677$$

(8) $$\mathrm{SK}\;\mathrm{method}:\;\ln\left(\frac{\beta }{T^{1.92}}\right)=\ln\left[\frac{k_{0}R}{g(\alpha )}{\left(\frac{E_{k}}{R}\right)}^{-0.92}\right]-1.0008\frac{E_{k}}{RT}-0.312$$

Following these isoconversional methods, the left term is linearly drawn against 1/T, and a series of straight plots are obtained for all the *α* levels considered. From the resultant slopes, the *E_k_* may be readily estimated over the entire range. As for the CR method, one may note that it has been initially proposed for kinetically evaluating pyrolysis data based on a single heating rate and later upgraded to analyze multi-heating-rate data. For this reason, the latter is sometimes renamed as model-free Kissinger–Akahira–Sunose (KAS) method [28]. However, in this article, the term of the CR method is preferred for studying co-pyrolysis of N-PE waste.

Considering that these methods are of approximate nature, Gao et al. [29] attempted to introduce two iteration terms to finalize the exact solution to temperature-integral function and then proposed a very good iterative program to obtain more accurate *E*_k_ values than the conventional isoconversional methods. The two equivalent iterative methods thus proposed, respectively corresponding to CR and FWO methods, are mathematically given below and named here it-CR and it-FWO in short:

(9) $$\mathrm{it}\text{-}\mathrm{CR}:\;\ln\left(\frac{\beta }{h(x)\cdot T^{2}}\right)=\ln\left[\frac{k_{0}R}{E_{k}g(\alpha )}\right]-\frac{E_{k}}{RT}$$

(10) $$h\left(x\right)=\frac{x^{4}+18x^{3}+86x^{2}+96x}{x^{4}+20x^{3}+120x^{2}+240x+120}$$

(11) $$\mathrm{it}\text{-}\mathrm{FWO}:\;\ln\frac{\beta }{H(x)}=\ln\frac{0.0048k_{0}E_{k}}{Rg(\alpha )}-1.0516\frac{E_{k}}{RT}$$

(12) $$H\left(x\right)=\frac{\exp(-x)\cdot h(x)/x^{2}}{0.0048\exp(-1.0516x)}$$

where *x* = *E_k_*/*RT*, and the iteration calculation may be briefly described as follows: (1) First, assuming *h*(*x*) = 1 or *H*(*x*) = 1, Equations (9) and (11) are respectively reduced to Equations (5) and (6), and ln(*β*/*T*^2^) vs. 1/*T* and ln *β* vs. 1/*T* can be plotted for each *α*, leading to the initial value of *E_k_*_1_ from the correspondent straight lines. Clearly, these values are identical to those from CR or FWO methods, respectively. (2) By applying *E_k_*_1_ to calculate *h*(*x*) and *H*(*x*), followed by drawing ln[*β*/*h*(*x*)*T*^2^)~1/*T* or ln[*β*/*H*(*x*)]~1/*T*, a new value of *E_k_*_2_ can be computed from the slopes of resultant straight lines. (3) Then, the calculation should be continued by replacing *E_k_*_1_ with *E_k_*_2_ until |*E*_ki_ − *E_k_*_i−1_| < 0.01 kJ/mol. (4) Finally, the latest value of *E_k_*_i_ could be considered as the exact activation energy. Accordingly, the *E_k_* can thus be yielded over the entire conversion range.

#### 2.3.2. Determination of lnk_0_ and f(a) Based on Compensation Effect

Pre-exponential factor ln*k*_0_ is another kinetic parameter, and it can be estimated according to the kinetic compensation effect between ln*k*_0_ and *E_k_*. Such effect has been recently addressed and exampled by Prof. Vyazovkin [30] for obtaining model-free ln*k*_0_ values provided that relatively accurate *E_k_* values are available from the model-free methods, and it can be linearly expressed as follows:

(13) $$\ln k_{0}=aE_{k}+b$$

where *a* and *b* are two characteristic compensation parameters, and then ln*k*_0_ may be calculated by substituting the calculated *E_k_* into Equation (13) once *a* and *b* are both available.

In this work, a new model-fitting method is proposed, according to the compensation effect, to determine ln*k*_0_ and *g*(*a*) together. For such purpose, the intercept of the final iteration based on the it-CR method, *I_f_*, for estimating *E_ki_* will be used here. From Equation (9), the following can be obtained:

(14) $$I_{f}=\ln\left[\frac{k_{0}R}{E_{ki}\cdot g(a)}\right]$$

Obviously, ln*k*_0_ and *g*(*a*) are both involved in Equation (14), and the following expression may be readily deduced from Equation (13):

(15) $$\ln k_{0}=I_{f}-\ln\left[\frac{R}{E_{ki}\cdot g(a)}\right]=aE_{ki}+b$$

With a known model *g*(*α*), *I_f_* and *E_ki_* obtained from the iteration, the ln*k*_0_ values can be estimated from Equation (15). Then drawing *I_f_* –ln{R/[*E_ki_*·*g*(*α*)]} against isoconversional *E_ki_* over the entire conversion range may yield a straight line for each *g*(*α*), and the performances of different *g*(*α*) models will be evaluated by comparing the linear correlation coefficient *R*^2^ values. The model *g*(*a*) with the *R*^2^ value of closest to or equal to 1.0 may be taken as the most appropriate one. At the same time, the two parameters of *a* and *b* will be also determined, and then the ln*k*_0_ values can be calculated by substituting *E_ki_* into Equation (13).

#### 2.3.3. Determination of Thermodynamic Parameters

Thermodynamic quantities for pyrolysis and oxidative thermal degradation of tennis string nylon-6 waste, including activation energy (*E_k_*), the change in entropy (Δ*S*), the change in enthalpy (Δ*H*) and the change in Gibbs free energy (Δ*G*), were calculated based on transition-state theory using the following expressions [31,32,33]:

Δ*S* = *R*·ln[(*k*_0_·*h*_P_)/(*e*·*k*_B_·*T*)] (16)

Δ*H* = *E*_k_ − *RT* (17)

Δ*G* = Δ*H* − *T*Δ*S* (18)

where *e* is the Neper number (2.7183), and *h_P_* and *k*_B_ represent Plank constant (6.626 × 10^−34^ J/s) and Boltzmann constant (1.381 × 10^−23^ J/K), respectively. Herein, ln*k*_0_ is the pre-exponential factor determined as above, and *E*_k_ is the activation energy from the iteration method.

## 3. Results and Discussion

### 3.1. FTIR Examination

**Figure 1:**
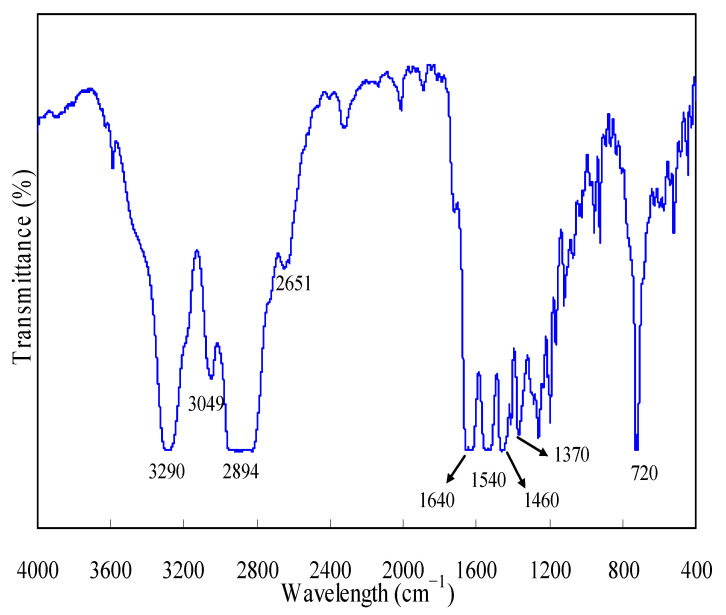
FTIR analysis results for N-PE waste.

Figure 1 shows FTIR measurements conducted for N-PE waste, and these spectra can be elaborated as follows. The absorption band peaked at 3290 cm^−1^ can be attributed to the stretching vibrations of hydrogen-bonded NH groups while the adsorption bands centered at 3049, 2894 and 2651 cm^−1^ can be assigned to the stretching vibration of the −CH_,_ −CH_2_ and −CH_3_ groups. Meanwhile, the band of 1620~1660 cm^−1^ can be considered as the stretching vibration of the amide II and CN groups of nylon-6, and the one over 1520~1560 cm^−1^ can be due to the vibrations of amide I, almost identical to those reported in the literature [34]. Furthermore, the bands around 1450~1470 and 1380~1360 cm^−1^ are respectively attributed to the bending vibrations of the −CH_2_ and −CH_3_ groups while the band around 710~730 cm^−1^ is probably due to the rocking vibration of the −CH_2_ group [35,36]. The above information tends to suggest there may be no strong interaction between nylon-6 and PE, even though they are co-present in the waste sample.

### 3.2. Pyrolysis Features of N-PE Waste

**Figure 2:**
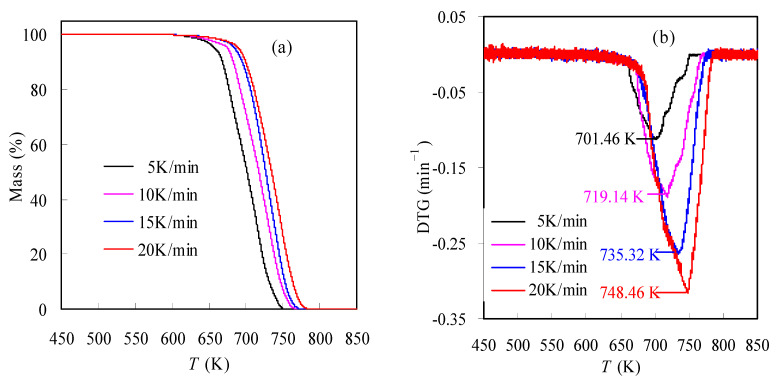
Pyrolysis TGA results (**a**) and DTG curves (**b**) of the N-PE waste under different heating rates.

Figure 2a graphically presents the TGA results of the N-PE waste sample determined in inert N_2_ under a constant ramping rate of *β* = 5, 10, 15 or 20 K/min. Clearly as seen, the T-dependent mass loss curve moves to a lower-temperature range as the heating rate *β* declines and vice versa. For example, the sample is seen to lose its mass in the range of 629.06~746.24 K at 5 K/min, but the mass loss range shifts upward to the range of 652.17~766.85 K at 15 K/min. Such a result is related to the resistance of the sample to heat transfer. Due to the poor thermal conductivity, the inside and outside of the sample may be evenly heated, and such a case appears to have a worse result as the heating rate goes up. Subsequently, the thermal resistance leads to the nonuniform mass transfer, and some components inside the sample cannot gasify promptly to respond to the rise in the heating rate, instead, leading to a temperature-shift compensation.

Figure 2b reflects the multi-heating-rate DTG curves of the N-PE waste, and one may note that each DTG curve only demonstrates one peak where the sample shows a maximum mass loss rate (d*α*/d*t*). Here, the temperature at the peak is named *T*_p_ for simplicity, resulting in one *T*_p_ for one *β*. Similar to those observed from the TGA results, the DTG curves are seen to heavily depend on *β*, and the curve will shift rightward to high-temperature domains as *β* increases. At the same time, the *T*_p_ value increases, and the peak becomes strong when increasing *β* from 5 to 20 K/min. More importantly, based on the one-peak features of DTG results, the waste sample may undertake one reaction stage during the pyrolysis process, although two different polymers are involved. Such finding is less frequently reported in publications since two polymers are physically blended by co-extrusion. A possible explanation for it is that the respective pyrolyses of nylon-6 and PE are strongly overlapped with each other, and further effort to confirm this will be made in a subsequent study. Thereafter, a single-reaction model might be assumed for depicting the kinetic degradation process. Such an assumption might be very helpful for obtaining the most appropriate mechanism function for the pyrolysis of N-PE pouch wastes.

**Figure 3:**
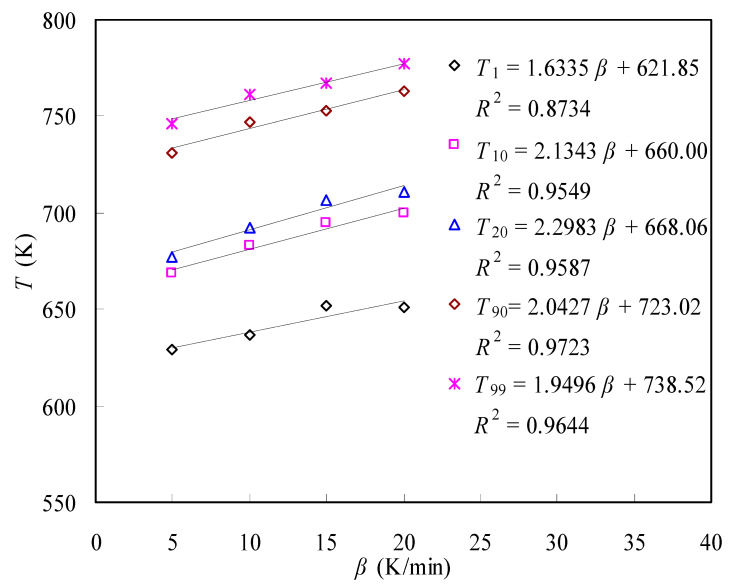
Relationship between heating rate *β* and specific pyrolysis temperatures.

To better examine the effect of the heating rate on the mass loss results, some specific temperatures are abstracted from TGA data and redrawn in Figure 3 for the N-PE waste. In Figure 3, *T*_1_, *T*_10_, *T*_20_, *T*_90_ and *T*_99_ are specified as the temperatures of 1, 10, 20, 90 and 99% mass conversion, respectively. As clearly seen, the relationship between *β* and these specific temperatures can be linearly quantified. The linearization parameters and the values of *R*^2^ (defined as the linear correlation coefficient) are all given in Figure 3 for reference. Expectedly, all specific temperatures demonstrate very good linear dependence on the heating rate, indicative of their linear increase with the heating rate used.

On the other hand, the *β* effect may be also considered by examining thermochemical performances in terms of the heat-resistance index (*HRI*) [19,37] and comprehensive performance index (*CPI*) [38,39] with respective mathematical forms as below:

(19) $$HRI=0.49\times [T_{5}+0.6(T_{30}-T_{5})]$$

(20) $$CPI=\frac{DTG_{p}\cdot DTG_{m}}{T_{i}T_{p}\Delta T}$$

where *T*_5_ and *T*_30_ are, respectively, the temperatures at the mass conversion of 5% and 30% while *T_i_* represents the temperature at the beginning of pyrolysis. *DTG*_p_ and *DTG*_m_ are the maximum mass loss rate (min^−1^) and the average mass loss rate (min^−1^), respectively. Meanwhile, Δ*T* means the temperature range in-between *DTG*/*DTG*_p_ = 0.5. Usually, the higher *CPI* value suggests a better thermochemical performance.

Table 1 presents some featured parameters for the pyrolysis of the N-PE waste. Based on *T*_5_ and *T*_30_ from TGA results, the *HRI* value can be evaluated according to Equation (2), and it is seen to slightly increase upward as *β* goes up, indicating the small influence of the heating rate on thermal resistance. In the meantime, *T*_p_, *DTG*_p_ and *DTG*_m_ are all directly acquired from DTG curves for four heating rates, the *CPI* value is then estimated according to Equation (2), and the results are given in Table 1 as well. By comparing the *CPI* values, it may be plausibly deduced that a higher heating rate tends to demonstrate better thermochemical features. A similar finding on the heating-rate effect was also reported in our earlier work [39,40].

**Figure 4:**
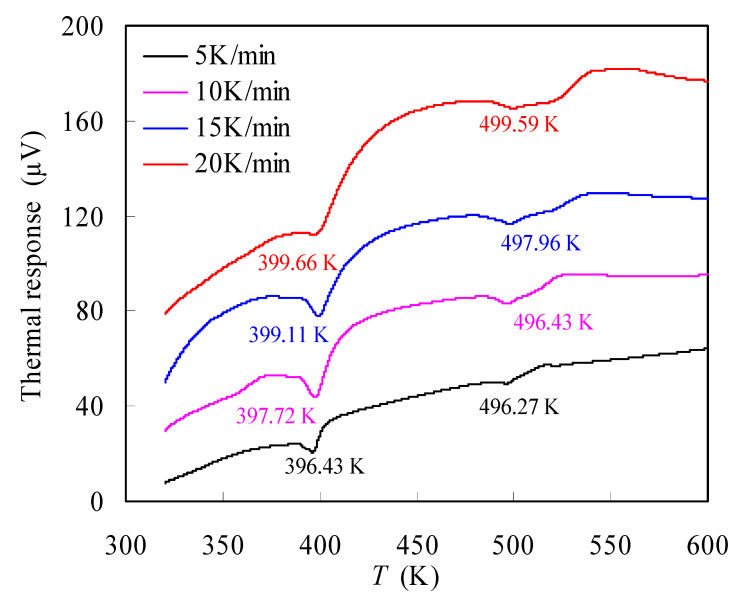
DTA results measured in N_2_ for N-PE waste.

Figure 4 shows the DTA results obtained over 5~20 K/min for the N-PE waste, where two endothermic melting valleys are observed for each heating rate due to the presence of two different crystalline structures. Obviously, they are, respectively, attributed to PE and nylon-6 crystals with melting points corresponding to 396~400 K and 496~500 K. These melting point values are consistent with those in the literature [7,41,42]. Unsurprisingly, two valley temperatures are seen to strongly depend on the heating rate as reflected by the change in the melting temperatures with the heating rate, similar to that observed for the *T_p_* value.

### 3.3. Kinetics Analysis of Thermal Degradation

#### 3.3.1. Determination of E_k_ with Various Kinetic Methods

**Figure 5:**
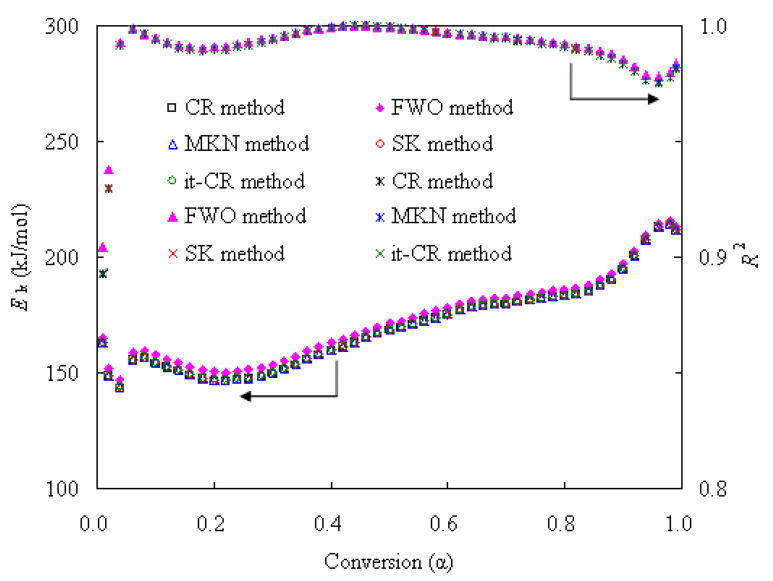
*E*_k_ calculated with various methods and calculation deviations *R*^2^ for pyrolysis of N-PE waste.

Generally, the *E_k_* can be readily computed via various model-free methods by linearly drawing an Arrhenius plot without the use of any assumed mechanism functions. After we collected the data (*α*, *T*) from TG results over the range of 0.01 ≤ *α* ≤ 0.99 for 5 ≤ *β* ≤ 20 K/min, the linear plots were drawn according to the four integral CR, FWO, MKN and SK methods. Figure S1 linearly presents the CR plots of ln(*β*/*T*^2^)~1000/*T*, FWO plots of ln(*β*)~1000/*T*, MKN plots of ln(*β*/*T*^1.884318^)~1000/*T* and SK plots of ln(*β*/*T*^1.92^)~1000/*T* for the N-PE waste. As clearly seen, the plots exhibit very good linearity, and such finding can be also supported by the linear correlation coefficient *R*^2^ values because these values are mostly very close to 1.0 as shown in Figure 5. Estimated by using Equations (5)–(8) from the slopes of resultant straight lines, the *E_k_* values are shown in Figure 5. In the meantime, the *E_k_* can be iteratively obtained with the it-CR or it-FWO methods, and the *E_k_* values thus achieved from the it-CR method are also given in Figure 5 along with the *R*^2^ values. It may be noted that the it-CR and it-FWO methods both led to identical results, and thus the results from the it-FWO method are not shown here.

As clearly seen, all the *α*-dependent *E_k_* curves show the same trend regardless of the model-free methods considered. However, the *E_k_* values from CR, MKN, SK and more accurate iteration methods are very close to each other for all *α* values, with a fluctuation of <0.5 kJ/mol, but substantially lower than those from the FWO method. Overall, the *E_k_* values from the FWO method are higher by 1.50~3.85 kJ/mol than those from the other methods. A careful examination shows that the *E_k_* values are 143.41~213.72 kJ/mol for the CR method, 146.96~215.22 kJ/mol for the FWO method, 143.78~214.04 kJ/mol for the MKN method, 143.74~214.05 kJ/mol for the SK method and 143.79~214.05 kJ/mol for the it-CR/it-FWO method, respectively. If compared to those reported for individual nylon-6 pyrolysis [11,12,13,14] or PE pyrolysis [15,16,17,18], one may find their *α*-dependent *E_k_* values are almost the same, and for this reason, the *E_k_* values for the co-pyrolysis of the N-PE waste are comparable to those of either nylon-6 or PE pyrolysis, indicative of the absence of the synergetic effect during the co-pyrolysis of the N-PE waste. After averaging, the *E_k_* values for the five methods are 169.68, 172.66, 170.01, 170.03 and 170.04 kJ/mol, respectively.

#### 3.3.2. Determination of g(α) and lnk_0_ Based on Compensation Effect

**Figure 6:**
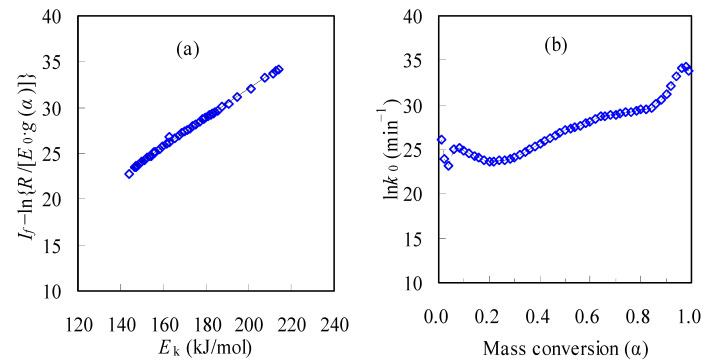
Linear compensation plots (**a**) and resultant ln*k*_0_ (**b**) for N-PE waste.

Apart from *E*_k_, the other two parameters ln*k_0_* and *G*(*a*) are determined by fully considering the compensation effect. It may be noted that one global reaction model is assumed here since there is only one peak in each DTG curve for the N-PE waste pyrolysis. Based on the it-CR method, the final intercepts of *I_f_* are also achieved for all *a* values, and then a number of reaction model *G*(*a*) functions [19,20] are scanned with the aid of Equation (15). Subsequently, by linearly correlating the compensation effect, i.e., $I_{f}-\ln\{R/[E_{k}\cdot g(\alpha )]\}$ versus $E_{k}$, the correlation coefficient *R*^2^ can be yielded for examining the linearity of all the correlations, and the closer to 1.0 the *R*^2^ value is, the better the linear relationship. Therefore, among the reaction models considered, the one with the *R*^2^ closest to 1.0 will be taken as the most appropriate function for describing the pyrolysis of the N-PE sample. Table 2 shows the results for over 24 specific functions scanned for the compensation effect consideration [19,20]. Based on the results in Table 2, one may deduce that the co-pyrolysis of the N-PE waste appears to undergo a reaction-order-based chemical process or the Avrami–Erofeev nucleation mechanism. Furthermore, the A_3/2_ model function, *g*(*α*) = [−ln(1 − α)]^2/3^, seems to be the best one among the 24 specific pyrolysis mechanism models because it led to the closest *R*^2^ value of 0.9981, as shown in Table 2. Obviously, such a mechanism model is not like the chemical reaction mechanism model reported for the pyrolysis of nylon–6 [11,12,13,14] or PE [15,16,17,18], suggesting that the co-pyrolysis of the nylon-6/PE blend may have involved mutual interference from individual pyrolysis, leading to the change in the pyrolysis mechanism. With *a* and *b* thus obtained, the ln*k_0_* values are calculated by substituting the *E_k_* values from the it-CR method into Equation (15) accordingly. These ln*k_0_* values are shown in Figure 6, along with the linear compensation effect from the A_3/2_ model. The ln*k_0_* values are seen to be *a*-dependent as well, like the *E_k_* reflected in Figure 5. The ln*k_0_* is observed to vary from 23.14 to 34.26 min^−1^ for the N-PE waste, and the *k_0_* values are correspondingly in the range of 1.12 × 10^10^~7.56×10^14^ min^−1^.

**Figure 7:**
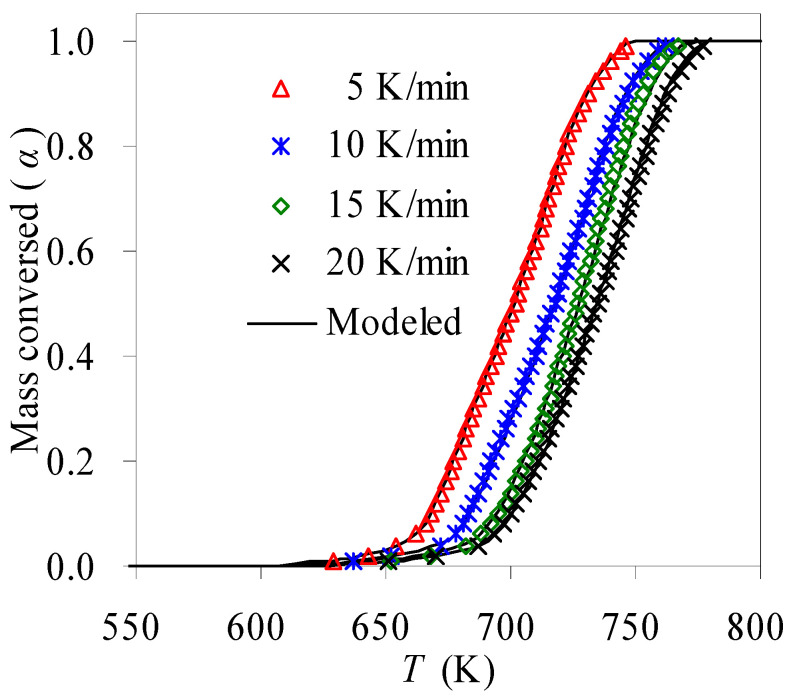
Predicated *α*~*T* curves and experimental results for N-PE waste.

With fully available kinetic triplets, theoretical predications for the pyrolysis process were thus made, and Figure 7 exhibits the predicted results along with experimental data. As can be seen, the A_3/2_ mechanism model, combined with *E*_k_ from the iteration method and ln*k*_0_ data from the compensation effect, excellently recast the *α*~*T* curves over the entire range of 0.01~0.99 for the N-PE waste because the data points abstracted for each *β* are nearly all collapsed on their respective prediction curves.

#### 3.3.3. Service Life Prediction

Lifetime information is very useful to properly consider a polymer for specific use requirements, and usually, the service time of various polymer-based products will be shortened under relatively high temperature conditions. Here, the lifespan of the N-PE waste was studied for thermal treatment consideration. Following the work earlier reported [31,43], it may be reasonably assumed that the plastic article cannot serve anymore when its mass loss approaches 5%, i.e., *α* = 0.05, and then the life span, *t*_s_, can be estimated with the following expression:

(21) $$t_{s}=\frac{g(\alpha )}{k_{p}}\exp\left(\frac{E_{p}}{RT}\right)$$

where *E_p_* is the activation energy evaluated according to Kissinger’s plot of $\ln(\beta /T_{p}^{2})$ versus 1/*T_p_*, and g(α) is taken to follow the first-order reaction with *g*(*α*) = −ln(1 − *α*) while *k*_p_ is the pre-exponential factor which is obtained as given below:

(22) $$k_{p}=\frac{\beta E_{p}^{}}{RT_{p}^{2}}\exp\left(\frac{E_{p}}{RT_{p}}\right)$$

**Figure 8:**
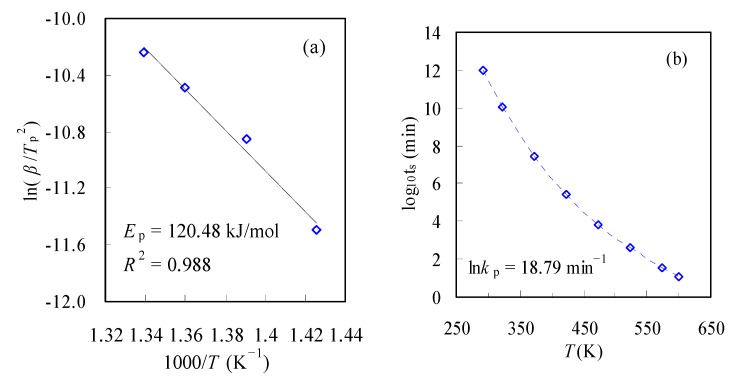
Kissinger’s plot of ln(*β*/*T*_p_^2^)~1/*T*_p_ (**a**) and lifetime predication (**b**) for N-PE waste.

Using the *T_p_* data, Kissinger’s plot can be drawn accordingly and is shown in Figure 8a. From the resultant line slope, the *E_p_* thus calculated is 120.48 kJ/mol, and clearly, it is considerably smaller than those obtained with model-free methods. Subsequently, the ln*k*_p_ value is estimated by using Equation (22) and varies very little with the change in the heating rate *β*. The ln*k_p_* averaged over four *β*s is 18.79 min^−1^. Thereafter, the lifetime prediction can be performed with the use of *E_p_* and ln*k_p_*, and the results are presented in Figure 8b.

As obviously seen, *t*_s_ is heavily relying on temperature, and it declines substantially with the rise in temperature. These findings agree with those reported in the literature [31,32]. According to the estimation, it may be noted that such packaging bags can withstand 100 °C very well for approximately 49.5 years, but the service time *t*_s_ at 150 °C will be reduced to about 0.5 years or 183 days. This information provides meaningful knowledge for selecting proper thermal conditions for applying polymer-based products.

#### 3.3.4. Thermodynamic Parameters

**Figure 9:**
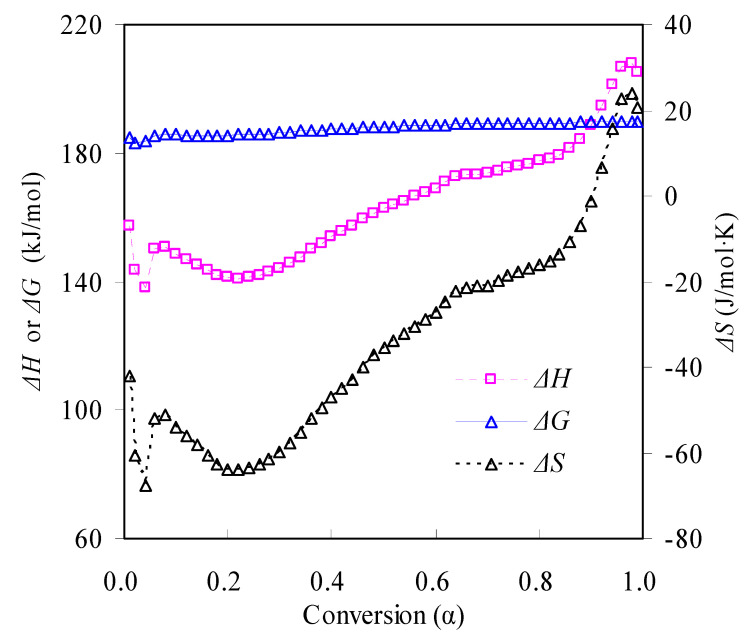
Thermodynamic parameters Δ*H*, Δ*S* and Δ*G* for the N-PE waste pyrolysis.

Thermodynamic analysis may be performed for the pyrolysis of the N-PE waste according to the transition state theory [31,32,33]. Accordingly, thermodynamic quantities in terms of Δ*G*, Δ*H* and Δ*S*, i.e., the enthalpy change, entropy change and Gibbs free energy change, respectively, were calculated using the *E_a_* values from the it-CR method and ln*k*_0_ from the linear compensation effect. These parameters thus calculated are presented in Figure 9. As can be seen, all the Δ*H* values are positive but *α*-dependent over the whole conversion range, indicative of an endothermic feature. Thus, external energy must be provided for conducting the waste pyrolysis. In contrast, the Δ*G* values almost remain unchangeable around 187.66 kJ/mol over 0.01 < *α* < 0.99. These positive Δ*G* values suggest a thermodynamically non-spontaneous process that requires the heat introduced for undergoing pyrolysis. Furthermore, as mass conversion progresses, the proceeding pyrolysis becomes less favorable as reflected by higher Δ*G* for higher *α* values. As also observed from Figure 9, most Δ*S* values, ranging from −66.66 to 23.83 J/mol·K, are negative during the pyrolysis process, indicating a transition from a disordered state to an ordered state according to the transition state theory. However, such ordering is rather dubious since high-molecular-weight polymers will crack into small molecules during high-temperature pyrolysis. Further work along this line is thus required and should be conducted to deeply understand the thermodynamics of pyrolysis.

## 4. Conclusions

In this work, the pyrolysis of N-PE pouch waste was studied non-isothermally under 5–20 K/min, and kinetic and thermodynamic analyses were thus conducted in detail. Some conclusions may be drawn as follows:

1. TGA results show that the co-pyrolysis of the N-PE pouch waste underwent one decomposition stage, indicating that the respective pyrolyses of nylon-6 and PE may overlap with each other. In the meantime, all specific temperatures of *T*_1_, *T*_5_, *T*_30_, *T*_p_ and *T*_99_ and pyrolysis performance parameters *CPI* and *HRI* are all found to increase with the heating rate.
2. The co-pyrolysis of the N-PE pouch waste was kinetically analyzed, and the *E*_k_ values from the isoconversional CR, MKN, SK, it-CR and it-FWO methods are very similar to each other, whereas the ones from the FWO method are fairly higher. For all these methods, the *E*_k_ values within 143~215 kJ/mol demonstrated strictly the same α-dependence.
3. The methodology of combining model fitting with the compensation effect was tried for seeking the pyrolysis mechanism together with the pre-exponential factor, resulting in the A_3/2_ model of *g*(*α*) = [−ln(1 − *α*)]^2/3^, or *f*(*α*) = 3/2 (1 − α)·[−ln(1 − α)]^1/3^ with the *k*_0_ range of 1.12 × 10^10^~7.56 × 10^14^ min^−1^ for the pyrolysis of the N-PE waste. With *E*_k_, *k*_0_ and *g*(*α*) thus obtained, the *α*-T curves were calculated and then demonstrated very satisfactory correlations against experimental results.
4. Thermodynamic parameters in terms of Δ*G*, Δ*H* and Δ*S* and the service lifetime predictions were estimated concerning the pyrolysis decomposition process of the N-PE waste, and the information provided helpful knowledge for applying polymer-based products and future waste disposal.

## Figures and Tables

**Table 1:** Some featured parameters for pyrolysis of N-PE waste.

Parameters	*β* (K/min)			
	5	10	15	20
*T*_5_ (K)	658.43	675.94	685.10	690.82
*T*_30_ (K)	685.38	701.12	714.17	719.59
*HRI* (K)	330.55	338.61	344.25	346.96
*T*_i_ (K)	600.61	602.75	601.72	597.74
*T*_p_ (K)	701.46	719.14	735.32	748.46
*DTG*_p_ (min^−1^)	−0.113	−0.189	−0.264	−0.315
*DTG*_m_ (min^−1^)	−0.016	−0.030	−0.038	−0.051
Δ*T* (K)	51.97	62.81	57.04	68.90
*CPI* (×10^10^ min^−2^·K^−3^)	0.82	2.07	4.02	5.27

**Table 2:** Some specific function g(a) scanned and calculation results.

No.	Symbol	*g*(*α*) Function	ln*k*_0_ = *a*·*E*_k_ + *b*		
			*a*	*b*	*R* ^2^
Chemical reaction equation					
1	F_1/3_	*g*(*α*) = 1 − (1 − *α*)^2/3^	0.1631	−1.3526	0.9931
2	F_1/2_	*g*(*α*) = 1 − (1 − *α*)^1/2^	0.1660	−2.0653	0.9934
3	F_2/3_	*g*(*α*) = 1 − (1 − *α*)^1/3^	0.1693	−2.9472	0.9938
4	F_3/4_	*g*(*α*) = 1 − (1 − *α*)^1/4^	0.1710	−3.4938	0.9940
5	F_3/2_	*g*(*α*) = (1 − *α*)^−1/2^ − 1	0.1909	−5.7914	0.9953
6	F_2_	*g*(*α*) = (1 − *α*)^−1^ − 1	0.2080	−7.6834	0.9938
7	F_3_	*g*(*α*) = (1 − *α*)^−2^ − 1	0.2477	−13.0338	0.9848
8	F_1_	*g*(*α*) = −ln(1 − *α*)	0.1768	−2.9720	0.9947
9	G_1_	*g*(*α*) = 1 − (1 − *α*)^2^	0.1481	1.8709	0.9932
10	G_2_	*g*(*α*) = 1 − (1 − *α*)^3^	0.1421	3.0684	0.9943
11	G_3_	*g*(*α*) = 1 − (1 − *α*)^4^	0.1381	3.8420	0.9951
Sigmoidal rate equation					
12	A_1/3_	*g*(*α*) = [−ln(1 − α)]^3^	0.2879	−23.0267	0.9080
13	A_1/2_	*g*(*α*) = [−ln(1 − α)]^2^	0.2323	−12.9993	0.9528
14	A_2/3_	*g*(*α*) = [−ln(1 − α)]^3/2^	0.2046	−7.9856	0.9763
15	A_3/4_	*g*(*α*) = [−ln(1 − α)]^4/3^	0.1953	−6.3144	0.9834
16	A_3/2_	*g*(*α*) = [−ln(1 − α)]^2/3^	0.1583	0.3705	0.9981
17	A_5/2_	*g*(*α*) = [−ln(1 − α)]^2/5^	0.1435	3.0445	0.9900
18	A_2_	*g*(*α*) = [−ln(1 − α)]^1/2^	0.1491	2.0417	0.9946
19	A_3_	*g*(*α*) = [−ln(1 − α)]^1/3^	0.1398	3.7129	0.9856
20	A_4_	*g*(*α*) = [−ln(1 − α)]^1/4^	0.1352	4.5486	0.9785
Other mechanism equation					
21	P_1/2_	*g*(*α*) = *α*^1/2^	0.1398	3.4122	0.9914
22	P_1/3_	*g*(*α*) = *α*^1/3^	0.1336	4.6266	0.9821
23	G_7_	*g*(*α*) = [1 − (1 − α)^1/2^]^1/2^	0.1437	2.4951	0.9929
24	G_8_	*g*(*α*) = [1 − (1 − α)^1/3^]^1/2^	0.1453	2.0541	0.9935

## Data Availability

The data presented in this study are available on request from the corresponding author.

## Supplementary Materials

The following supporting information can be downloaded at: https://www.mdpi.com/article/10.3390/ma16175738/s1, Figure S1: The Arrhenius linear plots of the N-PE waste with: (a) CR method, (b) FWO method, (c) MKN method and (d) SK method.
