# Supplementary material for: Kinetic and Thermodynamic Analyses of Co-Pyrolysis of Nylon-Polyethylene Pouch Wastes
Source: Materials (Basel). 2023 Aug 22;16(17):5738. doi: 10.3390/ma16175738 (PMC10488972; doi:10.3390/ma16175738)
Supplement: Supplementary file 1 [file materials-16-05738-s001.zip › materials-2516710-supplementary.pdf]

Supplementary materials

Kinetic and thermodynamic analyses of co-pyrolysis of nylon-polyethylene pouch wastes

Hai-bo Wan, Zhen Huang

Tianjin University of Commerce, Tianjin 300134, China

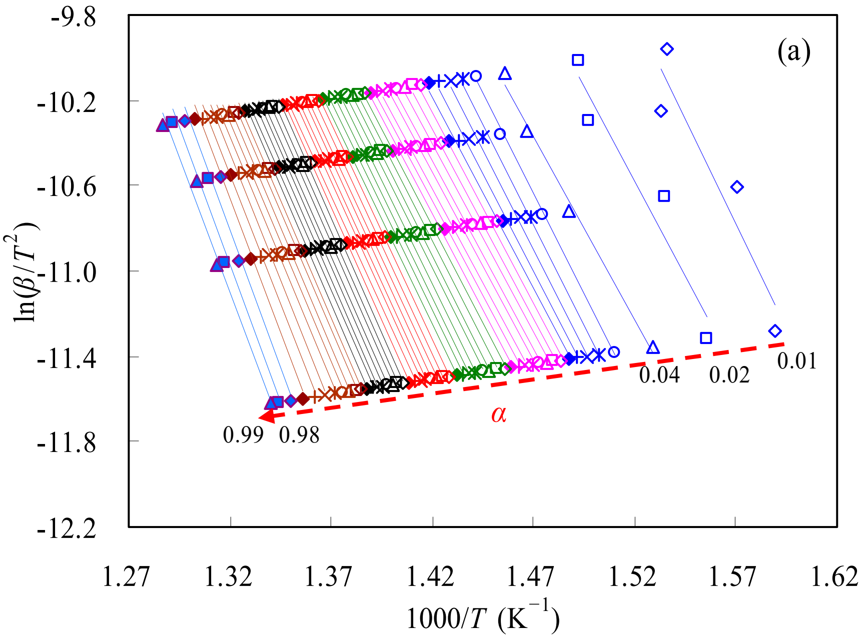
(a)
ln(β/T²)
1000/T (K⁻¹)
α
0.99 0.98
0.04 0.02 0.01
-9.8
-10.2
-10.6
-11.0
-11.4
-11.8
-12.2
1.27 1.32 1.37 1.42 1.47 1.52 1.57 1.62

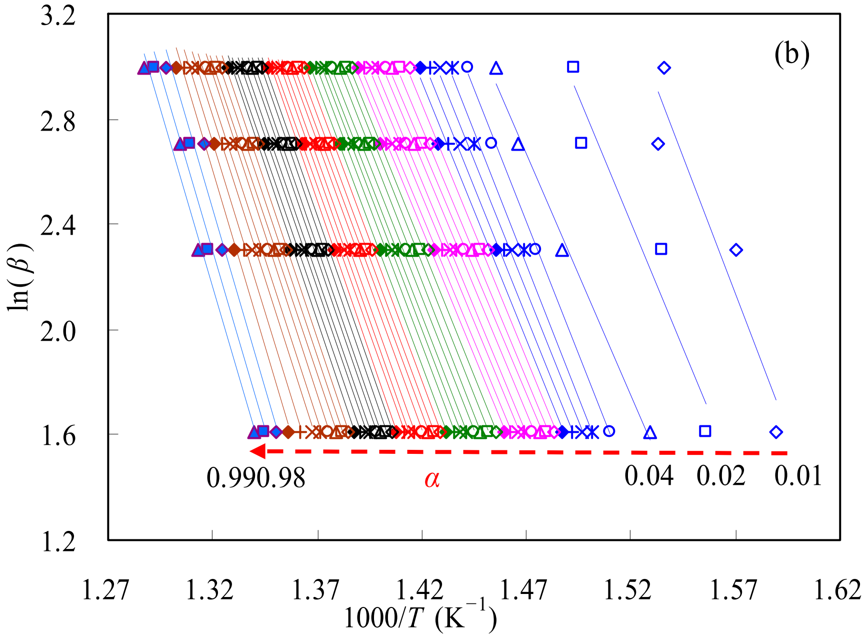
(b)
ln(β)
1000/T (K⁻¹)
α
0.990.98
0.04 0.02 0.01
3.2
2.8
2.4
2.0
1.6
1.2
1.27 1.32 1.37 1.42 1.47 1.52 1.57 1.62

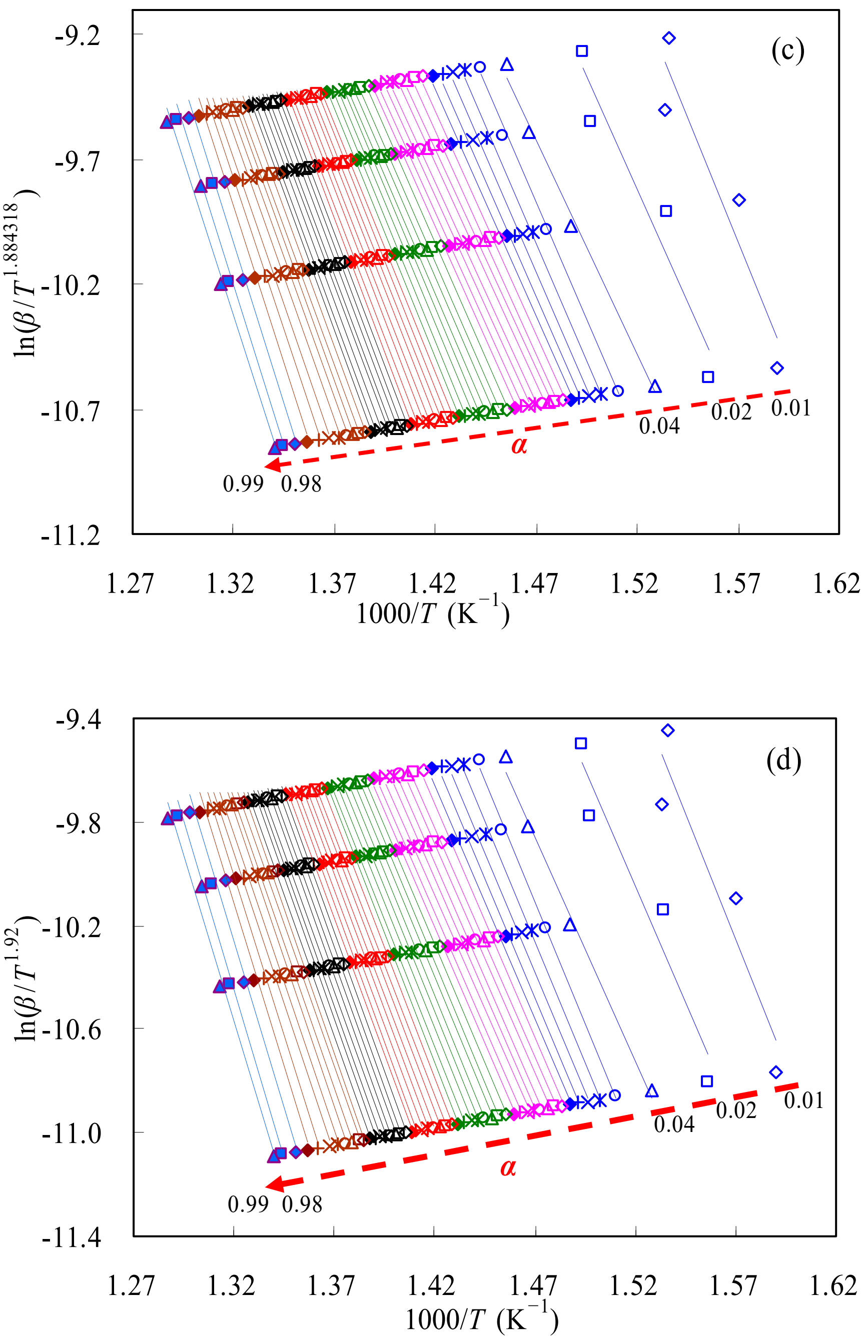

Figure S1. The Arrhenius linear plots of the N-PE waste with: (a) CR method, (b) FWO method, (c) MKN method and (d) SK method.
